# Potential of Wood Hemicelluloses and Their Derivates as Food Ingredients

**DOI:** 10.1021/acs.jafc.2c06449

**Published:** 2023-02-01

**Authors:** Felix Abik, Chonnipa Palasingh, Mamata Bhattarai, Shaun Leivers, Anna Ström, Bjørge Westereng, Kirsi S. Mikkonen, Tiina Nypelö

**Affiliations:** † Department of Food and Nutrition, University of Helsinki, P.O. Box 66, Helsinki 00014, Finland; ‡ Department of Chemistry and Chemical Engineering, Chalmers University of Technology, Gothenburg 41296, Sweden; § Department of Bioproducts and Biosystems, Aalto University, P.O. Box 16300, Espoo 00076, Finland; ∥ Faculty of Chemistry, Biotechnology and Food Science, Norwegian University of Life Sciences, Ås 1430, Norway; ⊥ Helsinki Institute of Sustainability Science (HELSUS), University of Helsinki, P.O. Box 65, Helsinki 00014, Finland; # Wallenberg Wood Science Center, Chalmers University of Technology, Gothenburg 41296, Sweden; □ Department of Bioproducts and Biosystems, Aalto University, Espoo 00760, Finland

**Keywords:** xylans, mannans, emulsions, novel foods

## Abstract

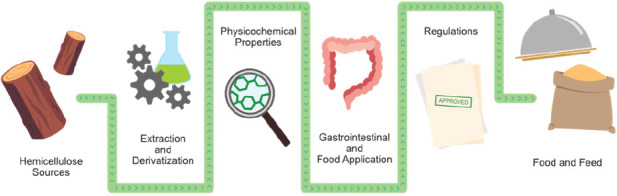

A holistic utilization of all lignocellulosic wood biomass, instead of the current approach of using only the cellulose fraction, is crucial for the efficient, ecological, and economical use of the forest resources. Use of wood constituents in the food and feed sector is a potential way of promoting the global economy. However, industrially established food products utilizing such components are still scarce, with the exception of cellulose derivatives. Hemicelluloses that include xylans and mannans are major constituents of wood. The wood hemicelluloses are structurally similar to hemicelluloses from crops, which are included in our diet, for example, as a part of dietary fibers. Hence, structurally similar wood hemicelluloses have the potential for similar uses. We review the current status and future potential of wood hemicelluloses as food ingredients. We include an inventory of the extraction routes of wood hemicelluloses, their physicochemical properties, and some of their gastrointestinal characteristics, and we also consider the regulatory route that research findings need to follow to be approved for food solutions, as well as the current status of the wood hemicellulose applications on that route.

## 1 Introduction

Motivated by the ever-increasing human population, new food ingredients are being discovered and developed at a remarkable rate. These new food ingredients must fulfill various quality criteria to be widely accepted for consumption, including safety, technological, nutritional, economical, and environmental aspects. There is also an increasing societal demand for clean-label food products that are derived naturally from biomass instead of relying on synthetic materials.^1^ While there have been technological efforts to reduce food waste and make the food industry more circular, as well as the booming development of plant-based food, it is also possible to obtain edible material from seemingly inedible sources.^2^ One such promising alternative source is lignocellulosic biomass, in particular, wood, which contains hemicelluloses that can be developed into functional food ingredients. From our perspective, wood hemicelluloses present a large opportunity to be developed into and marketed as naturally derived food ingredients, fulfilling the current industrial demands of clean label food and feed products.

Hemicelluloses compose a remarkable family of polysaccharides found in plant cell walls. In food systems, they are long known as important dietary fibers, which can be extracted from various sources. One promising source of such extractable fibers is wood biomass. Hemicelluloses can be liberated from wood biomass as polymeric, oligomeric, and monomeric fractions with coextraction of noncellulosic compounds. This wide range of possible extractable grades of material leads to diversity in molar mass and physicochemical properties as well as opening a window toward numerous applications, including those as potential food ingredients. Complete exploitation of wood hemicelluloses allows resource-efficient use of wood biomass and minimizes volumes of waste streams currently generated from biomass processing within the forest sector.

This Review focuses on presenting the status of wood hemicelluloses and their derivates as food ingredients. Although the structure and properties of wood hemicelluloses as well as chemical reactions for their derivatization are largely established, currently there are only few academically demonstrated commercial and industrial solutions, and it has proven difficult to progress the scientific demonstrations into emerging applications. Thus, a description of the route to regulatory and legislative approval, the essential step from demonstration to food applications, is also included. Many of the academic studies deal with hemicelluloses in emulsions, and therefore the science-to-technology approach requires an understanding of the mechanisms behind hemicelluloses at interfaces; therefore, research within that area is included in this Review. This Review also covers wood and wood-like hemicelluloses in general, common derivatization methods that are potentially acceptable for food applications, and their key physicochemical properties.

Readers are also encouraged to refer to previous reviews on the structure and solubility of hemicelluloses,^3^ isolation, structural characterization, and potential applications of bamboo hemicelluloses,^4^ recent developments and challenges of obtaining plant-based nanomaterials from plant residues,^5^ applications for functional foods,^6^ bioethanol,^7^ and oligosaccharide production,^8,9^ and polysaccharide degradation by microbial^10^ and technological^11^ processing. Hemicelluloses in packaging materials have been reviewed recently^12^ and are therefore excluded from this Review.

## 2 Wood and Wood-Like Hemicelluloses

**Figure 1:**
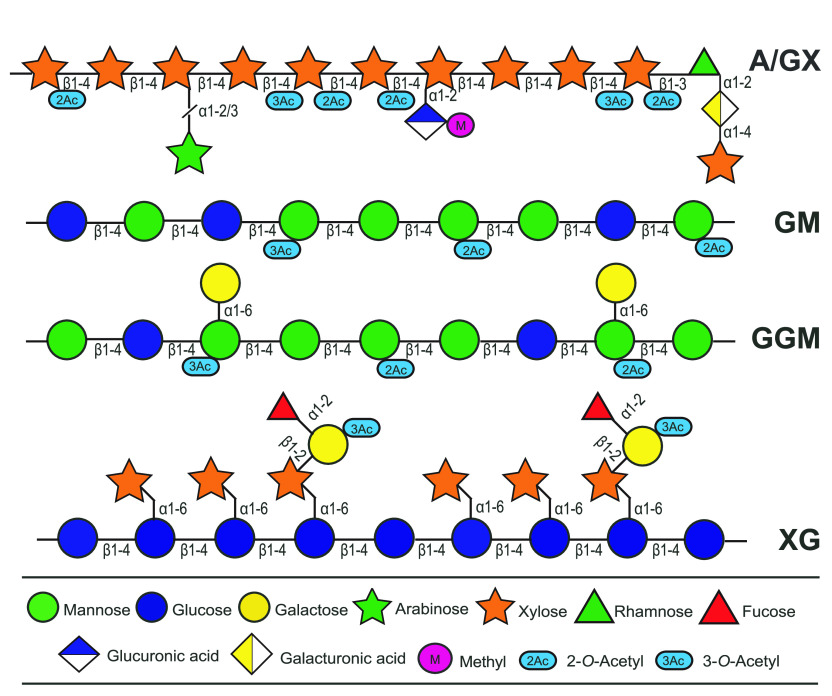
Structure of wood hemicelluloses, illustrating the backbones and side chains of the different types of hemicellulose. A/GX indicates that GX may exist in wood with or without arabinose residues. α1-2, α1-4, α1-6, β1-2, β1-3, and β1-4 indicate the type of glycosidic bonds; α1-2/3 in A/GX indicates that the arabinose residue may attach to hydroxyl groups at position 2 or 3 of the xylose residue. 2-*O*-Acetyl and 3-*O*-acetyl indicate that acetylation may occur at the hydroxyl position 2 or 3 of the respective carbohydrate residue. The symbols are sketched according to Varki et al.^17^

The hemicelluloses that can be liberated in industrially relevant yields from wood are xylans and glucomannans (GM), containing substituents such as arabinose (Ara), glucuronic acids (GlcA), and galacturonic acids (GalA) in xylans, and galactose (Gal) in mannans. Both types of polymers are also acetylated to various extents in the native state in the plants. Approximately 15–35 wt % of hardwood and 30–32 wt % of softwood macromolecular composition is made up of hemicelluloses.^13−15^ Softwoods such as Norway spruce (*Picea abies*) and white spruce (*Picea glauca*) are rich in galactoglucomannan (GGM) (16–17% of dry wood weight) with some arabinoglucuronoxylan (AGX) (8–10%), while hardwood, for example, paper birch (*Betula papyrifera*), is rich in glucuronoxylan (GX) (15–30%) with some GM (1–2%).^14,15^ Xyloglucans (XG) make up 20–25% of the cell wall in dicotyledonous angiosperms, 2–5% in grasses, and 10% in softwoods.^16^ However, due to their lesser abundance in wood, XGs are typically not considered as one of the main hemicellulose streams from wood, and as such we focus our discussions on GGMs and GXs. Details regarding the structural features of both softwood and hardwood hemicelluloses have been extensively described by Sjöström^14^ and Ebringerova et al.,^3^ and an overview of the structure of these hemicelluloses is presented in Figure 1.

Because of the central and versatile role of hemicelluloses in the cell wall structure, wood is not the only source of xylans and mannans. In particular, xylans can be extracted as arabinoxylans (AX) from various types of nonwood biomass, such as sugar cane bagasse,^18^ straw,^19^ corn fibers,^20^ cereal brans, and grass,^21^ while mannans can be isolated as galactomannan (GalM) from seeds and beans^22^ or GM from konjac tubers.^23^ Many of these hemicelluloses, such as GalM isolated from seeds, guar gum, and locust bean gum, entered the food system several decades ago.^22^ While there are structural differences between seed-based mannans and xylans as compared to those isolated from wood (such as the degree of acetylation for both, and glucuronic acid for the latter), they share a similar backbone and structural motifs. Because of the structural similarities, we therefore hypothesize that hemicelluloses from wood can also be considered as food ingredients. However, including a food ingredient from new sources, even if it is already in use from an existing source, requires approval and permission from the European Commission (EC).^24^ The procedure is described later in this Review; acquiring the permission is currently a gatekeeper to the commercial use of wood hemicelluloses as food ingredients. Fortunately, wood as the source of biomass is not an intrinsic limitation. Cellulose derivatives obtained from wood are considered safe food and feed ingredients and are commonly used across industries (E460, E461, E462, E464, E465, E468, etc.).^25^

### 2.1 Extraction of Hemicelluloses from Wood and Other Biomasses

There are multiple ways one can liberate hemicelluloses from wood biomass using hot water extraction of wood chips at neutral, acidic, and alkaline conditions, hot water,^26−28^ neutral-, acidic-, and alkaline extraction of wood chips, delignified wood biomass, or wood pulp.^29,30^ New methods are continuously being developed to improve the efficiency, sustainability, and selectivity of the extraction process. In fact, improving the efficiency while reducing the cost of the extraction and purification processes may be the key to facilitating the exploitation of wood hemicelluloses when it comes to their extraction at an industrial scale.

The extraction conditions have been reported to have a large effect on the chemical composition of the hemicelluloses. Perez et al.^31^ reported that, for softwoods, near neutral or acidic extraction yields GGM with low residual lignin content, while alkaline extraction conditions lead to mixtures of GGM and AGX. Acidic treatment of hardwoods has been reported to produce mixtures of AGX and GGM, while the fractions obtained in alkaline conditions were comprised of AGX. In general, neutral and acidic conditions result in GGM fractions, while alkaline conditions favor xylan liberation.^26^

Additionally, extraction conditions determine the purity of the isolated hemicellulose. Ebringerova and Heinze^32^ described a process for liberating xylan from the cell wall matrix. In a process targeting xylan, the hardwood species of choice is first delignified with acidic NaClO_2_ or preferably with H_2_O_2_/NaOH. This leads to separation of biomass into a solubilized extract and a residue; the former requires purification if the other constituents from the plant material are not desired in the final product. The residue is subjected to alkaline extraction and results in a water-insoluble xylan extract. The purification step is considered demanding; nevertheless, fractionation of the hemicellulose and lignin components is most often desired. However, for certain applications, complete purification of the extract might not be necessary as was demonstrated for lignin-rich spray-dried birch GX extract obtained by pressurized hot-water extraction (PHWE) that was found to be a more efficient emulsion stabilizer than its ethanol-precipitated counterpart containing less lignin.^33^

Subjecting wood chips to hydrothermal treatments results in oligomeric fragments, either as part of a prehydrolysis step to generate dissolving pulp or exclusively to isolate the hemicelluloses as oligosaccharides or short polysaccharides. The most commonly explored method is the hydrothermal hydrolysis of the wood biomass, either with the addition of a small amount of acid for prehydrolysis^34,35^ or without in the case of autohydrolysis, where the acetyl groups are released as acetic acid, subsequently acidifying the mixture.^36,37^ During the hydrolysis process, hemicellulose chains are cleaved into smaller fragments, which are then liberated into the hydrolysate. The hydrolysate may also contain several byproducts, including lignin, released as the wood structure is compromised. Furfurals from degraded pentoses and acetic acid from the cleaved acetyl groups of the hemicellulose may also be present. The production of these byproducts, in particular, furfurals and acetic acid, is increased as the extraction process becomes more severe.^38,39^ However, it is possible to eventually separate them from the hydrolysate to obtain the hemicelluloses and other components at a considerable purity.^40−42^ Other hydrothermal extraction methods include steam explosion and microwave-assisted hydrothermal extraction, each with specific release and degradation kinetics as compared to conventional hydrothermal hydrolysis processes.^43−46^

Ultimately, dealing with materials obtained from nature requires an understanding that the structure of the extracted materials will primarily be determined by the source. In the case of hemicelluloses, plants intrinsically have a source-specific dominant hemicellulose and carbohydrate composition, as well as various nonhemicellulose components that may be coextracted. In addition, it can be generally considered that hydrolysis leads to short chains, while hot water and alkaline extraction are more likely to generate polymeric hemicelluloses. With those points taken into consideration, a set of suitable extraction methods and conditions are chosen to achieve the desired product, as the extraction conditions also affect the physicochemical properties of the extracts. In essence, to obtain a specific type of hemicellulose with certain desired properties, one must choose carefully both the source of biomass and the suitable extraction method.

### 2.2 Common Derivatizations Potentially Acceptable for Food Applications

**Figure 2:**
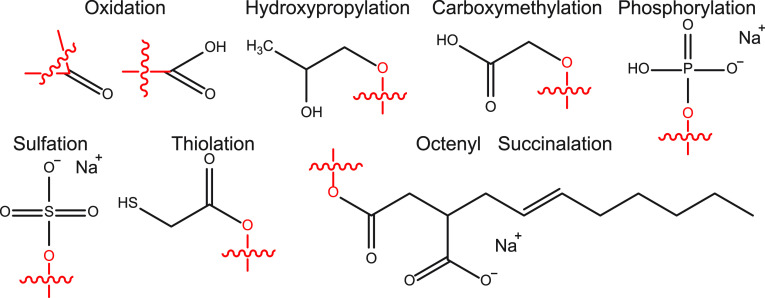
Structures of the common derivatizations performed on other polysaccharides potentially acceptable for food applications. Red wavy lines indicate the connection of the substituent groups to the polysaccharide chain.

Although hemicelluloses have a range of naturally occurring derivatizations in the form of acetyl, methyl, and acidic groups as well as extensive branching,^47^ further chemical modification and cross-linking may be required to achieve various specific effects in food.^48^ We begin here by considering the available derivatizations for polysaccharides by inspecting examples of routinely chemically modified polysaccharides to illustrate the possible array of chemical synthesis tools, applicable also to wood hemicelluloses. Common derivatizations are shown in Figure 2. While reactions such as cross-linking and derivatization are accepted, particularly for starches, highly defined inclusion levels must still be adhered to so as to meet strict purity criteria for use in food.^49,50^

Starch, consisting of the polysaccharides amylose (α-(1 → 4)-glucoside) and amylopectin (α-(1 → 4)-glucoside with α-(1 → 6)-glucoside branching),^51^ can be readily modified, and numerous forms of derivatized starch have been approved for use as food additives.^50^ Common, approved chemical derivatizations of starch include etherification ((carboxy)methylation,^52^ also used in food-grade cellulose; hydroxypropylation^53^), esterification (acetylation,^54^ phosphorylation,^55^ and (octenyl)succinylation^56^), and oxidation.^57^ Cross-linking, enzymatic, and physical methodologies have also been routinely applied and extensively studied.^58^ Such modifications can induce functionalities including lowering the viscosity, increasing the stability and/or clarity, improving the binding and moisture retention, gelation under controlled conditions, and increasing the crystallinity.^59^ Pectins (E440), a class of galacturonans commonly used as food and beverage ingredients, are routinely de-esterified to alter the degree of methylesterification.^60^ They can also be amidated,^61^ and amidated pectin (E440b) is currently the only approved modified pectin used in the food industry.^62^ Nevertheless, numerous derivatized pectins have been assessed, particularly for their potential use in a medical setting (thiolated,^63^ sulfated,^64^ and oxidized^65^ forms).

Many of the derivatizations and cross-linking strategies already approved for other polysaccharides have been fundamentally applied to both xylans and mannans. Beechwood xylan, a common target in xylan studies, has been shown to be successfully carboxymethylated^66^ and homogeneously sulfated.^67^ Other hemicellulose xylans, such as those derived from corn straw, have been phosphorylated and characterized with regards to functionality for further uses.^68^ Spruce mannan was oxidized^69^ and carboxymethylated^70^ as a pretreatment technique to examine the effect on hydrolysis yields and emulsification, respectively. Other sources of mannan, such as that from yeast, have been studied extensively and chemically carboxymethylated, phosphorylated, and sulfated.^71^

## 3 Key Physicochemical Properties of Wood Hemicelluloses and Their Derivatives

### 3.1 Solubility of Extracted and Derivatized Hemicelluloses

Polysaccharide solubility governs their technological properties as emulsifiers and stabilizers, and potentially other functionalities resulting from colloidal interactions. Polysaccharide dissolution is a complex balance of chain–chain interactions (inter/intramolecular) and chain–solvent interactions. Intrinsic polysaccharide characteristics such as molar mass, degree of side-chain substitution, type of substituents, and solvent conditions such as pH, ionic strength, and temperature alter these interactions.^72^ It is especially relevant in food items, where the system is composed of multiple ingredients that may influence or be influenced by the inclusion of wood hemicelluloses. We review here the key items relevant to wood hemicellulose solubility and include contributions not only relevant to the food field, to give a comprehensive picture of their solubility.

Inter- and intrachain bonding, as well as side-chain substitutions, provide both physical and chemical properties that can be controlled for dissolution. Branched polysaccharides are generally easier to dissolve in water, such as the case for water-soluble arabinogalactan;^73^ however, other types of substitution also affect the solubility. Wood GGMs’ solubility is affected by acetyl and Gal substituent units. Xu et al.^74^ showed that deacetylated GGMs formed macroscopic aggregates, while Parikka et al.^75^ observed how enzymatic oxidation of Gal units in GGMs enhanced intramolecular interactions via hemiacetal bonds. Mannans are often partially acetylated and are highly soluble in water as compared to alkaline extracted xylans, where the labile acetyl groups undergo deacetylation, which reduces their water solubility.^76^ To preserve the native acetyl groups, extraction can be performed with nonaqueous solvents, such as DMSO.^77^ However, using DMSO as the extraction solvent may be problematic when considering food-related applications due to potential residual solvent.

The presence of lignin and lignin-derived compounds, which are usually coextracted with hemicelluloses to a varying degree during hot-water extraction, is reported to modify GGM solubility. Bhattarai et al.^78^ showed that crude GGM extracts obtained via PHWE method were less soluble in water than the same crude extracts purified via ethanol-precipitation. It was shown that aqueous suspensions of crude GGM extracts contained soluble hemicelluloses of 10 kDa molar mass and insoluble submicrometer fractions. Using cryo-transmission electron microscope imaging and small-angle X-ray scattering analysis, it was shown that some crude GGM extracts were partly consolidated to nano- and submicrometer structured objects resembling lignin nanoparticles.

Studies by Kishani et al.^79^ suggest that water is a poor dispersion medium for native grades of GGM. The research referred to GGMs recovered from different extraction approaches, namely high pressure heating (100 °C, 5 min), hot-water extraction (60 °C, 3 h) of wood chips, and effluent from a thermomechanical pulp (TMP) plant. Regardless of the extraction and postextraction process, such as the removal of extractives and lignin impurities, all extracts displayed poor to very poor solubility in water, rendering aggregates up to 1000 nm. The molar masses of the GGMs obtained using these extraction procedures are seemingly higher than those from the PHWE process (21–66 kDa vs 10 kDa), which can render solubility differences. However, differences in the chemical structure of hemicelluloses from different recovery procedures also play a crucial role in multifactorial aspects affecting wood hemicelluloses’ solubility.

As some native hemicelluloses are sparingly soluble in water, solvents such as aqueous alkaline solutions, DMSO,^80^ and cosolvent systems^77^ are used to dissolve or solubilize hemicelluloses. Additionally, chemical modification of hemicelluloses, for example, esterification, oxidation, and etherification, have been used to add functional groups, while modification of the backbone structure has also been employed to improve the hemicellulose–water interaction.^29,81^ The degree of substitution was observed to play a key role in the solubilization of carboxymethylated GGM as the hydrodynamic volume increased as a function of degree of substitution.^70^ Carboxymethylation and dihydroxypropylation improved the water solubility of xylan, demonstrated by the reduced turbidity of the solutions as compared to unmodified xylans.^82^ The solubility of xylans is similarly affected by their side groups and acetylation.^3,83^ Kishani et al.^79^ reported the hydrodynamic radii of AGX to be <10 nm at a 0.1 wt % concentration, increasing to >60 nm at a 1.5 wt % concentration. For etherified xylans, aggregation at low degrees of substitution has also been observed, while an increased degree of modification resulted in reduced aggregation.^84^ For oxidized hemicelluloses, the aggregation has been reported to be correlated to concentration and inversely correlated to the degree of modification.^29,81^

### 3.2 Degree of Polymerization (DP)

Degradation during the liberation processes defines the molar mass of the extracted products. The wood hemicellulose extracts commonly possess a heterogeneous molar mass and exist as mixtures of polymers and oligomers. The length of a linear polymer chain often has a tremendous effect on potential end use applications. Short chains are useful when low viscosity, fast solubilization (or even depolymerization) during use is sought, or when aiming at high inclusion levels as, for example, in high fiber containing products. On the other hand, longer chains are a requisite for applications where, for example, high viscosity or emulsification properties are desired as well as engineering applications, where entangled networks form stronger materials.^85^ Use in packaging materials requires film formation^86^ and barrier-relevant properties^87^ that small molecules often lack. Consequently, hemicelluloses incorporated in packaging films^88−91^ utilize long-chain polysaccharide fractions or include the hemicelluloses in a matrix of higher molar mass polymers.^84^ Because of the aforementioned aspects, it is of key importance to have a precise DP determination.

Hemicellulose insolubility in water complicates molar mass analysis because, due to clustering, the molar mass distribution assigned to the single polysaccharides remains unelucidated. Therefore, gel permeation chromatography, also known as size-exclusion chromatography, combined with universal calibration or multiangle light scattering techniques are commonly used for the molar mass determination. This size-based fractionation method uses porous columns to separate the analytes on the basis of hydrodynamic volume. Solubility is therefore crucial to characterize the individual polysaccharide chains and to avoid erroneous estimation of molar mass values. Hence, solubility is likely to be one of the prominent reasons for the discrepancies in molar mass values reported in the literature. Insolubility also reduces the column’s lifetime as well as increases the risk of capillary blockage. The latter issue has been addressed to a large extent by an alternative method named asymmetric-flow field-flow fractionation (AF4), which does not rely on a stationary column,^78^ but where a disposable ultrafiltration membrane is used for separation, eliminating the risk of column blockage. AF4 has been applied in the analysis of molar mass and characterization of aggregates from spruce GGMs.^78^ The residual lignin in hemicelluloses is also thought to interfere with molar mass analysis as the autofluorescence of lignin interferes with the scattered laser light, hampering the analysis and thus leading to an overestimation of molar mass values. The fluorescence interference can be partly reduced by applying narrow bandwidth filters that are transparent only for the applied wavelength for light scattering as discussed previously.^93^

When the molar mass of the hemicelluloses has been determined, it is possible to obtain the DP value by dividing the average molar mass value by the molar mass of the anhydrous monosaccharide unit. Typical denominator values are 162 and 132 for neutral hexoses and pentoses, respectively, although the value can be different when the hemicelluloses’ backbone involves charged or oxidized monosaccharides or contains noncarbohydrate substituents. Using number-average molar mass will subsequently yield number-average DP, and, in the same way, weight-average molar mass will yield weight-average DP.^92^

**Figure 3:**
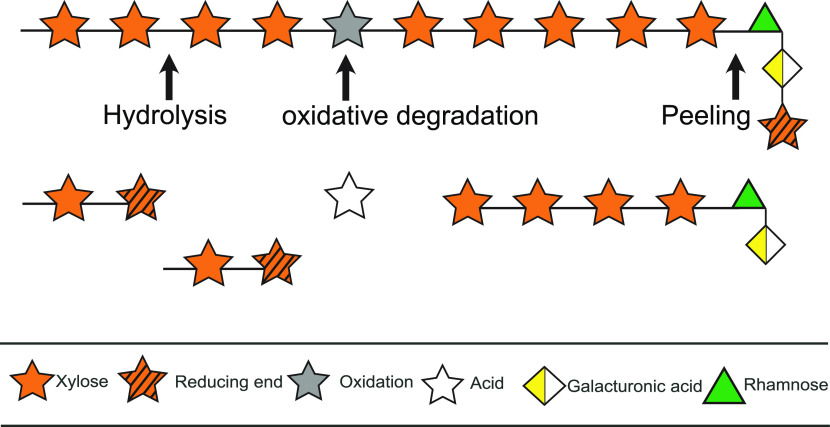
Mechanism of wood polysaccharide (shown for xylan) degradation during pulping. Adapted with permission from Sixta et al.^15^ Copyright 2006 Wiley.

A range of molar masses from 5 to 85 kDa and 4 to 67 kDa are reported for polysaccharide fractions of mannans and xylans, respectively (Table 1). Deliberate extraction that leads to larger molecules has been achieved by incorporating microwave irradiation of raw wood material in aqueous alkaline solution, instead of traditional heating.^26^ The atmospheric pressure and relatively mild conditions (temperature <100 °C and pH 5) led to materials generated with a molar mass up to 67 kDa from spruce and demonstrated less degradation than the hydrothermal treatment. The formation of oligosaccharides (3–10 residues)^94^ is often a consequence of degradation in the process, for example, by oxidation, or due to autohydrolytic effects caused by the release of acetic acid.^43,95^ For alkaline pulping processes, the degradation mechanisms of wood carbohydrates have been characterized by hydrolysis, oxidative peeling, and endwise peeling (Figure 3). In alkaline media, the reducing xylose group is removed by a β-elimination, which leads to a reducing galacturonic acid end group.^96^ The reactivity of carbohydrates in the prevalent kraft pulping process is steered by the morphology, crystallinity, and degree of polymerization.^15^ Within carbohydrates in wood biomass, cellulose is of a higher degree of polymerization and is more resistant toward alkaline media, and hence degrades to a lesser extent than hemicelluloses. Furthermore, alkaline conditions at elevated temperature, such as in cooking of pulp, will induce alkaline deesterification resulting in deacetylated hemicelluloses. Degradation of hemicelluloses may also take place in post processing treatments such as chemical modification. Oxidation with sodium periodate and subsequent reduction has been reported to lead to degradation that is manifested in a lowered molar mass.^81^

**Table 1:** Molar Mass (Weight-Average) of Liberated Wood Hemicellulose Polysaccharides Reported in the Literature

mannans	*M*_w_ (kDa)	refs
hot-water extraction of wood biomass	5–85	(27,28,97)
hot-water extraction from TMP process water	48–53	(27,28)
alkaline treatment	56	(98)

xylans	*M*_w_ (kDa)	refs
hot-water extraction	4–8	(27,99)
alkaline extraction	8–67	(26,29,30,100,101)

### 3.3 Assembly at Solid–Liquid Interfaces

Understanding how polysaccharides and their clusters assemble at interfaces is central to unlocking potential uses in food and feed. A solid–liquid interface is present, for example, when the polysaccharides of interest interact with other macromolecule components in food or the mucosal layer in the gastrointestinal tract. In these events, adsorption or lack of adsorption (depletion) may take place. At the solid–liquid interface, an adsorption occurs for two main reasons: the adsorbate has a strong affinity toward a surface and/or a poor interaction with the solvent. This means that the structure and chemistry of the adsorbate along with the surface morphology and adsorption conditions (e.g., adsorbate concentration, pH value, and salt concentration) govern the overall adsorption. Surface-sensitive techniques with nanoscale resolution such as quartz crystal microbalance with dissipation monitoring and surface plasmon resonance are typically employed for investigating solid–liquid adsorption, and both techniques have been successfully implemented in solid–liquid adsorption studies involving hemicelluloses.^102−104^

Hemicelluloses and their derivatives have been adsorbed on biopolymer surfaces in studies that mainly use cellulose as the substrate biopolymer. The focus of this Review is on food ingredients and wood hemicelluloses, but examples of other uses and nonwood hemicelluloses are added here to enrich the description. Molar mass, conformation, substituting units, and degree of substitution of hemicelluloses are factors that determine the adsorption. The influence of the molar mass of XG,^105,106^ xylans,^107^ and oxidized xylans^29^ has been studied; it was found that a higher molar mass increased the adsorption of hemicelluloses on the cellulose surface, and this was partly assigned to their tendency to aggregate. However, substituting units attached to the hydroxyl moieties of the polysaccharide backbone may also influence the adsorption behavior, both for native and for chemically modified hemicelluloses. In the case of AX, a low amount of arabinose substitutions decreased the water solubility as unsubstituted parts tended to interact with other unsubstituted components and form aggregates, benefiting liquid–solid adsorption.^102,108^ However, a high degree of arabinose substitution could hinder adsorption because AX is better solvated and hence remains in the solution instead of adsorbing to the solid interface. Acetyl groups increased the steric hindrance on xylan and consequently decreased the self-association of xylans, hampering its adsorption on the cellulose surface.^104,107,109^ Fucose units on XG side chains were found to influence the interaction with cellulose positively; however, the molar mass and conformation of XG were found to be more important.^110,111^ On the contrary, in the case of GGM, the amount of Gal units had a greater impact on the interaction with cellulose rather than the molar mass.^112^

When charges are involved in the adsorption, surface charge, polymer charge, and ionic strength become decisive adsorption parameters,^113^ which is relevant in our discussion as several wood hemicelluloses contain GlcA residues. Lee et al.^114^ reported on the adsorption of cationic-modified xylan on model cellulose surfaces. They found that low charge-density xylan was adsorbed as a thicker and more viscoelastic layer as compared to its high charge-density counterpart. Additionally, the ionic strength of the solvent environment played a role in the adsorption behavior, where a high salt concentration increased the adsorbed mass of high charge-density xylan due to screening of the charges, resulting in a thicker adsorbed layer. Moreover, the charge of the solid substrate also participates in the adsorption process. Uncharged, oxidized xylans were reported to be adsorbed less on negatively charged cellulose as compared to an uncharged cellulose substrate, indicating an influence of electrostatics despite only the substrate being charged.^29^

Surface morphological characteristics, such as surface area and porosity, can also influence adsorption. This aspect is especially relevant in food processing steps that involve membranes, as the adsorption of the hemicelluloses on the surface may lead to membrane fouling.^115^ In general, a large accessible surface area has a positive effect on adsorption; however, adsorption on internal surfaces, for example, in porous media, is also affected by the size of the pores, as the adsorbate may only adsorb at the internal surface if it can penetrate the pores.^107^ Benselfelt et al.^116^ studied the effect of cellulose crystallinity on XG adsorption. XG did not only bind on the surface but penetrated the cellulose film. Therefore, the morphology of the substrate, for example, that of the food stuff or surfaces in the gastrointestinal tract, may affect the adsorption of wood-derived hemicelluloses.

### 3.4 Assembly at Liquid–Liquid Interfaces

Emulsions constitute an integral part of the food industry in the form of beverages and oil-containing foods. Aside from their adsorption at the solid–liquid interface, wood hemicelluloses are known to adsorb at the liquid–liquid interface, leading to the physical stabilization of liquid and semisolid emulsions. The emulsion stabilizing capability has been investigated for hemicelluloses obtained from different extraction methods and purities,^27,117,118^ as well as chemically modified derivatives.^70,119−121^ As wood hemicelluloses are known to have low viscosity both in solution and as emulsions,^74,122^ the emulsion stabilizing capability has been attributed to interfacial activity (i.e., their ability to lower the interfacial tension and to assemble at the oil–water interface).^122,123^ Understanding this assembly and adsorption at the oil–water interface would allow for the generation of emulsions with tailor-made stabilities for various applications, utilizing the many different types of wood hemicelluloses.

**Figure 4:**
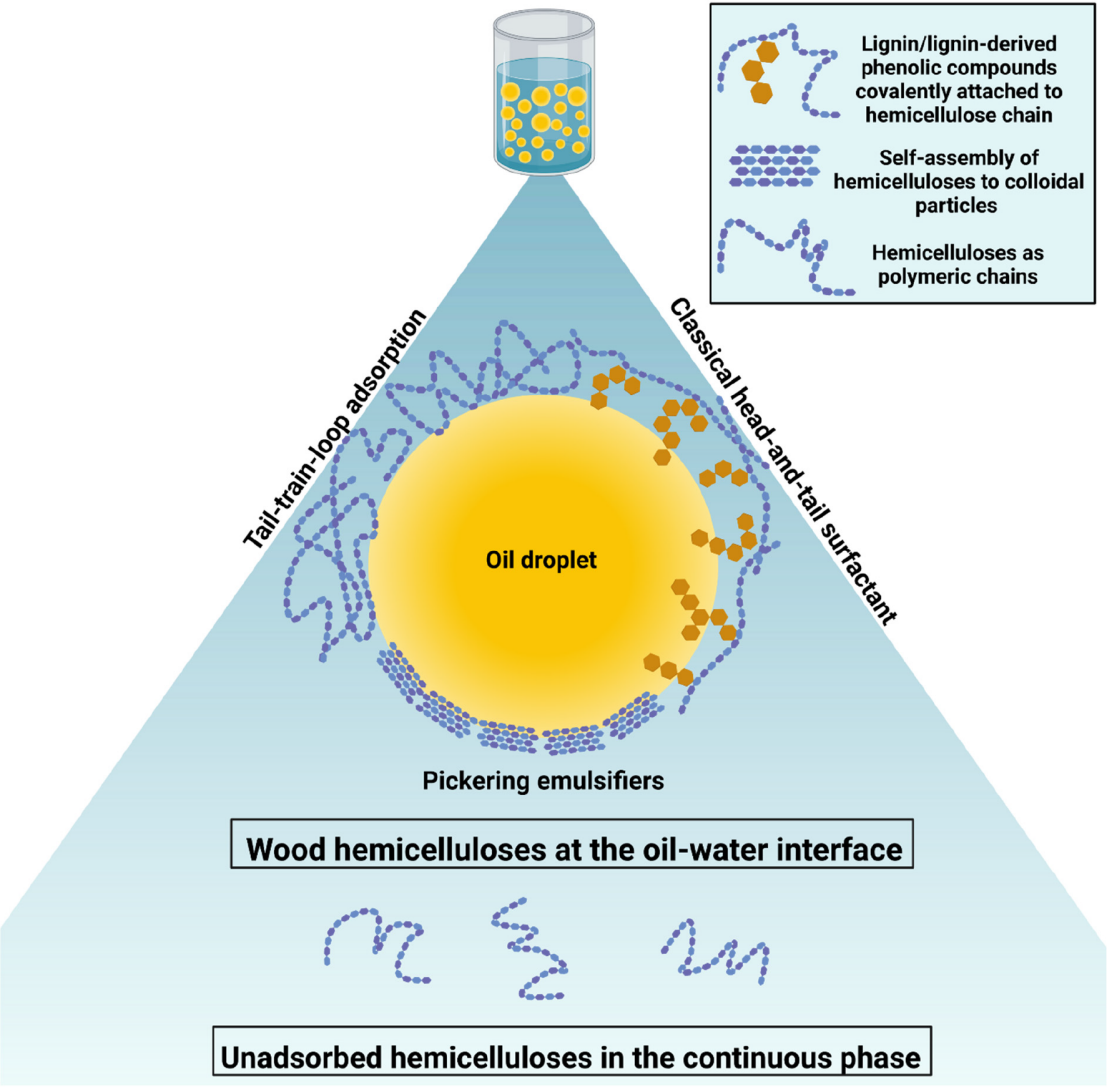
Adsorption modes of wood hemicelluloses at the liquid–liquid interface. Yellow spheres indicate oil droplets in an emulsion, while the blue background indicates the aqueous phase. Blue hexagons represent carbohydrate units, while brown hexagons represent lignin residues. The illustration also indicates that there may be a fraction of the hemicelluloses that are unadsorbed and are simply dispersed in the continuous phase. Created with BioRender.com.

There are three possible models of hemicellulose adsorption at the oil–water interface, an overview of which is illustrated in Figure 4. The first model follows the classical surfactant model, where the hemicellulose molecule is divided into a hydrophilic head and a hydrophobic tail. The hydrophobic tail anchors the entire molecule to the oil surface, while the hydrophilic head faces the aqueous phase.^124^ The hydrophobic moiety can be composed of residual lipids, proteins, or other hydrophobic materials.^125^ For example, it is well established that the protein-rich fractions in gum arabic are responsible for its interfacial activity,^126,127^ which consist of proteoglycans and lipids associated with the protein.^128^ For wood hemicelluloses, this model is particularly prominent in describing the interfacial activity of hemicelluloses that contain lignin–carbohydrate complexes (LCC). The covalent bond between lignin and hemicelluloses indicates that the lignin moiety could act as a hydrophobic anchor to the oil phase, while the aqueous phase-facing hemicellulose moiety could impart steric stability.^129,130^ Evidence of surfactant-like anchoring was provided by partitioning of adsorbed and unadsorbed hemicelluloses from model emulsions, followed by semiquantitative 2D nuclear magnetic resonance spectroscopy studies of the fractions.^131^ LCC species were found to be present in both fractions, with benzyl ether lignin-carbohydrate bonds (present in unpurified and nonpolar fractions of both PHWE GGM and GX) found exclusively bound to the oil–water interface. This finding explains the increased stability of emulsions stabilized by unpurified PWHE hemicelluloses as compared to the partially purified extract.^130^

The second model of adsorption details how a polymer chain adsorbs following the tail-train-loop conformation at the oil–water interface, such as in methylcellulose (MC). MC is a cellulose derivative in which some of the hydroxyl moieties are methyl-etherified, altering its inter- and intramolecular interactions as well as imparting interfacial activity. Following this model, the hemicelluloses would be adsorbed as random coils with loops that associate preferentially between the two phases.^132^ There have been previous studies that modified wood hemicelluloses into amphiphilic polymers by grafting hydrophobic moieties, both through esterification^120,133,134^ and through etherification^119,135^ of the hydroxyl groups, with the aim of altering the interfacial activity of the hemicelluloses in a similar manner by which cellulose is modified into MC. The results of those studies exhibited an increased surface activity of the hemicelluloses and evidenced consequent self-assembly. Naturally, wood hemicelluloses are acetylated, which contributed to reducing the extent of hydrogen bonding, subsequently decreasing their tendency to aggregate, and thus increasing water solubility;^136^ as such, it is plausible to assume that they might adsorb at the oil–water interface in a way similar to MC. However, there has been limited investigation into the correlation between the degree of acetylation of a given hemicellulose and the interfacial activity.

The third mode of adsorption is commonly known as Pickering stabilization, which requires solid particles or clusters of particles termed Pickering emulsifiers. Pickering emulsifiers adsorb at the oil–water interface, not as dissolved molecules, but as solid particles. The particles are considered to be adsorbed irreversibly at the oil–water interface due to the high free energy barrier associated with their desorption.^137^ There have been many explorations into the use of polysaccharide-based solid particles as Pickering emulsifiers.^138,139^ It is possible to prepare nanocrystals with xylan extracted from birchwood kraft pulp^140^ as well as nanoparticles from xylan and xylan derivates.^141−143^ Xylan nanocrystals from sugar cane bagasse have been successfully utilized as Pickering emulsifiers,^144^ which might indicate that wood-derived xylan nanocrystals have the potential to be used similarly. Additionally, insoluble clusters of alkali-extracted xylans from birch and beech also demonstrated efficient stabilization properties.^47^ Nevertheless, it is worth noting that, in a hemicellulose-stabilized emulsion, there is a possibility that nonpolysaccharide solid particles are also responsible for the stabilization. As mentioned previously, spruce GGM extracts containing lignin particles^145^ contributed to emulsion stabilization.^138,146^

In addition to their mode of adsorption, another interesting question to address regarding wood hemicelluloses’ adsorption at the liquid–liquid interface is the fate of the aggregates (in which they typically exist in aqueous systems), as the hemicelluloses are adsorbed at the oil–water interface. Despite documented evidence that wood hemicelluloses form aggregates in aqueous solutions/dispersions,^78,147^ there has only been limited information on whether the same form of assembly exists at the interface, especially those that are not adsorbed as Pickering emulsifiers. For example, previous studies of PHWE hemicellulose-stabilized emulsions indicated that both the hemicellulose and the phenolic fractions are adsorbed at the oil–water interface and contributed to both physical and chemical stability of the emulsion,^130,145^ and that the benzyl ether-type LCC is preferentially adsorbed at the interface as compared to phenyl glycosides and gamma esters.^131^ While those are valuable insights, the available data did not offer any indications regarding their assembly at the interface, as the adsorbed species had to be extracted prior to analysis and their conformation may potentially have been compromised. Considering their association with lignin, inspiration could be drawn from the investigation into the adsorption of gum arabic, which is known to be associated with protein species and form assemblies in its aqueous solution.^148,149^ Neutron scattering investigations of gum arabic-stabilized emulsions indicated that aggregates, present in its aqueous solution, dissociate at the oil–water interface to form an interfacial network that contributed to emulsion stability.^150,151^ Hence, it may be intriguing in the future to apply direct characterization methods for emulsion interfaces, such as neutron scattering^152^ and atomic force microscopy.^153^

## 4 Wood Hemicelluloses as Food Ingredients

### 4.1 Gastrointestinal Behavior of Hemicelluloses

Complex dietary glycans can affect the gastrointestinal tract in a number of ways, including influencing the microbiota composition. The gut microbiome is well-known to affect host well-being, with the preponderance of several bacterial species either positively or negatively correlated to anorexia,^154^ obesity and diabetes,^155^ and colorectal cancer,^156^ among others. The structural compositions of wood-derived xylans and mannans resemble other complex dietary fibers found in a variety of foods that stimulate beneficial bacteria and are therefore likely to be good candidates as alternatives to dietary fibers used today.

One prominent role of xylans and mannans as dietary fibers lies in their fermentability by the gut microbiota. More detailed knowledge about individual microbes’ potential to degrade specific complex carbohydrates present in food stuffs has recently emerged. Several detailed studies on complex degradation machineries in bacterial representatives from *Bifidobacterium*, *Firmicutes*, and *Bacteroidetes*(10) have been reported for xylans^157,158^ and mannans.^159−162^ This knowledge further enables rationally modifying the fibers to be preferentially digested by the microbe of interest, also known as microbiota-directed fiber (MDF). The use of MDF in food and feed has been investigated in several in vivo studies with positive results; specifically, xylans and mannans have been shown to stimulate growth of probiotic targets like *Bifidobacterium*,^163,164^*Roseburia intestinalis*,^157^ and *Faecalibacterium prausnitzii*.^156,164,165^ However, understanding the mechanisms of how different bacteria interact and affect their surroundings is still limited. For example, the observed effects might have arisen either directly due to the bacteria targeted by MDF or indirectly, from the proliferation of other microbial species that benefit from the MDF-targeted bacteria. This secondary “butterfly effect” may, in some cases, be more important than the direct effect of the MDF itself.^162^ As such, there are still challenges in demonstrating the beneficial effect toward the host in using xylans and mannans as MDFs. Fortunately, no adverse effects have yet been observed in the in vivo studies, and, as mentioned above, both wood-based hemicelluloses promoted the growth of probiotic target bacteria.^160,162,164^

### 4.2 Food Emulsions

Emulsions are indispensable in modern food product formulations, and a rising consumer demand for food products with all-natural, minimally processed ingredients has increased the global demand of functional polysaccharides from natural sources to act as emulsion stabilizers. Because of their interfacial activity, wood hemicelluloses have attracted interest as possible food emulsifiers and stabilizers. Spruce GGMs have exhibited both abilities in oil-in-water emulsion systems in many studies.^117,122^ Mikkonen et al.^123^ showed that GGMs promoted the long-term stability of emulsions more effectively than both gum arabic and corn fiber gum, which are commonly used hydrocolloids in commercial food formulations. It was also noted that the emulsion stabilization mechanism of cereal and seed-based hemicelluloses differs from that of wood-based hemicelluloses. Aqueous GGM and GX solutions exhibit a much lower viscosity and do not significantly modify the rheological properties of emulsions’ continuous phases at concentrations below 8 wt %;^122^ however, as elaborated on in the previous section, recent studies have proven the central role of residual lignin in their emulsifying capability. In bamboo xylans, coextracted proteins together with lignin contributed to the surface-active property and formed stable soy oil-in-water emulsions.^166^ Hemicellulose–protein complexes have also been formed to improve their emulsification abilities; for example, soyhull hemicelluloses conjugated with soy protein via the Maillard reaction have exhibited improved emulsifying properties.^167^

However, the stability of an emulsion is not only limited to physical stability against phase separation, but also the chemical stability of the oil-soluble compounds loaded in the oil phase. By the virtue of the lignin and lignin-derived phenolic groups, spruce GGM extracts acquire antioxidant capabilities and protect the dispersed oil phase against lipid oxidation.^130,168^ GGM was used to formulate a functional, drinkable yogurt with cod liver oil^169^ within this concept. Hemicelluloses have been used as stand-alone stabilizers or in conjunction with other polysaccharides such as gum arabic,^170^ providing a method to protect fragile bioactive components in various food matrices.

The presence of lignin and other impurities, however, might become a hindrance with respect to consumer acceptance of hemicelluloses. Pure hemicelluloses have a neutral flavor profile (similar to gum arabic); however, sensory analysis of yogurt supplemented by emulsions made with wood hemicelluloses indicated that a higher lignin content correlates to a more pronounced wood flavor.^171^ While no consumer acceptance test has been conducted thus far, the woody flavor might not necessarily be unwanted; it is known that flavors derived from wood are desirable in food items aged in wooden barrels such as alcoholic beverages^172^ and wine vinegar.^173^ Therefore, instead of a hindrance, it might open new avenues for woody flavors in foods.

### 4.3 Animal Feed

As established in the previous section, hemicelluloses are indigestible polysaccharides fermented by the gut microbiota. Spruce GGMs and birch GXs have exhibited this potential during in vitro^164,174^ and in vivo experiments in animal models, such as sheep,^175^ pigs,^162^ and mice.^176,177^ It is therefore implied that wood hemicelluloses could be applicable for use in animal feeds as well. The earliest known commercial product from wood hemicelluloses used in animal feed was Masonex, a hardwood hemicellulose extract produced by mild hydrolysis of wooden hardboard. It was included as a binding agent for pellet feeds. Masonex was found to be indigestible by broiler chickens^178^ and protected protein feed from degradation in the rumen.^179^ Over the years, more details on the effects of adding wood hemicelluloses to animal feed have been discovered. For example, mice fed softwood hemicellulose supplementation experienced a lower level of cholesterol, free fatty acid, and bile acids in their serum lipid composition as compared to the control group fed without supplementation, implying a possible cardiac protective property.^177^ In addition, spruce GGM may impart beneficial effects toward chronic prostate inflammation due to observed changes in the gut microbiota composition.^180^ Ruminants may also benefit from the addition of hemicelluloses in their feed, where it has been documented to enhance cellulose digestibility,^181,182^ improve lactation in dairy cows,^181^ and modify methanogenesis, which can potentially reduce methane emissions.^183^ While there are still knowledge gaps to be filled, such potential should be further explored.

### 4.4 Regulatory Items

Before entering commercial use as food ingredients, wood hemicelluloses must go through the premarket approval from food regulatory bodies specific to geographical locations where they will be marketed and distributed. The process of gaining premarket approval varies from one country or region to another. For example, in the United States (U.S.), food manufacturers may follow a process that relies on assessment independent of the federal agency, Food and Drug Administration (FDA), while the European Union (EU) follows a process regulated by the EC, facilitated by the European Food Safety Agency (EFSA), that provides independent scientific opinions on the safety of food. In this section, we will cover existing regulatory frameworks in the EU and the U.S.

**Figure 5:**
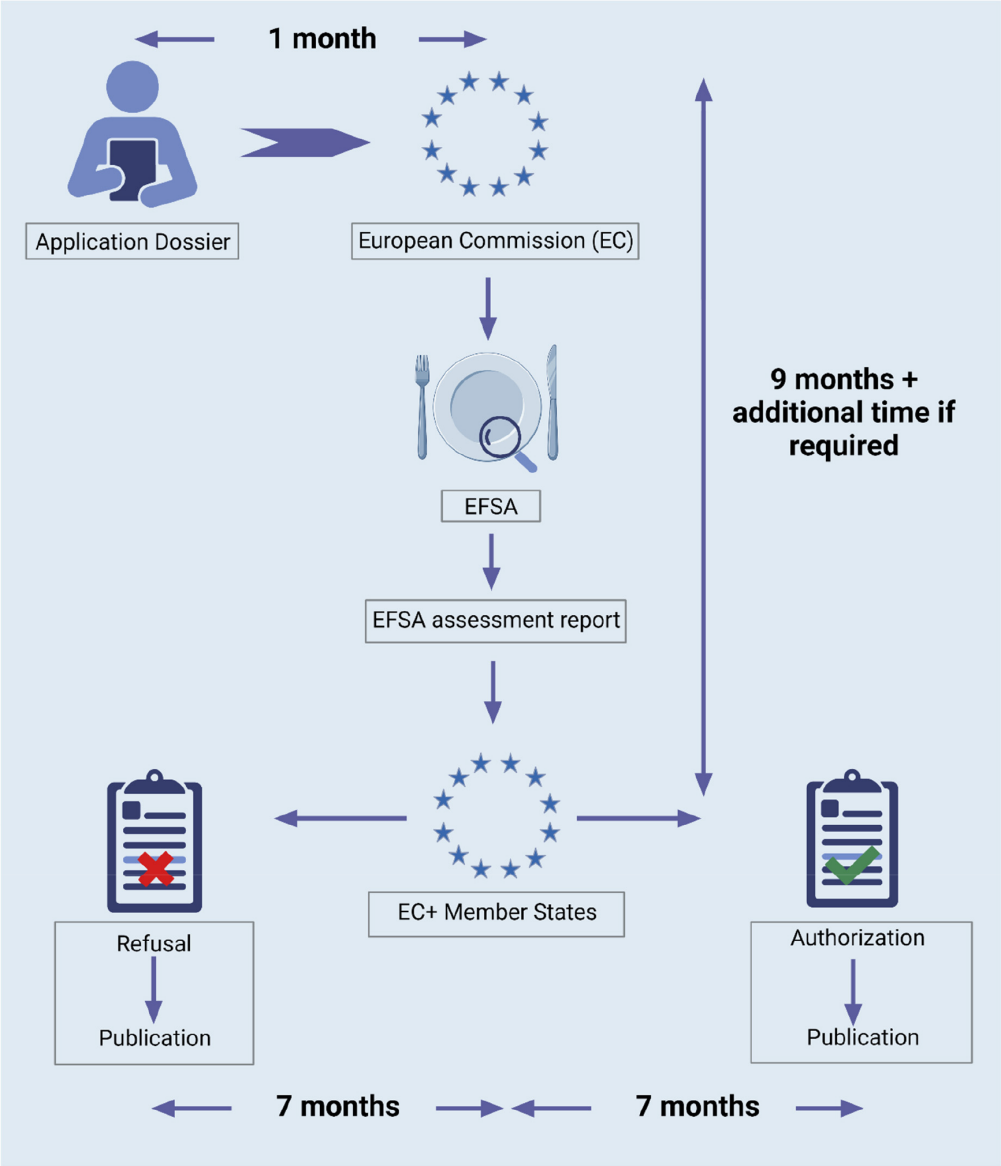
General novel food premarket approval summarized route in the EU. Created with BioRender.com.

Any food that was not consumed to a significant degree prior to May 1997 within the EU falls under the category “novel foods” pursuant to Regulation (EU) 2015/2283.^24^ It also includes food from new sources, new substances used in food, and new ways and technologies for producing food. Application for the approval of such a substance or food requires the submission of a dossier on the substance, which must include its identity and nutritional information. Required information in the dossier includes compositional data including stability; production process; toxicological data including genotoxicity, subchronic, chronic, reproductive, and developmental toxicities; absorption, distribution, metabolism, and excretion (ADME); proposed uses; usage levels; and anticipated intake including precautions and restrictions.^184^ After a complete submission of the information, a detailed risk assessment is performed by the EFSA as per EC mandate, which will take around 9 months provided no additional data are required from the applicant. When the risk assessment is adopted, the application reaches the postadoption phase where EFSA publishes the scientific output and EC drafts an implementation act. The complete process of authorization of a novel food substance, which requires a risk assessment, can take up to two years^185^ from the submission of the application (Figure 5).

While the molecular structure and the technological functions of wood hemicelluloses are known, few studies have focused on nutritional aspects and toxic contaminants, or side products formed during processing. Moreover, chemical and microbiological characterization of the intended substance pertaining to shelf life should be provided or addressed, and, when the novel food is expected to be used as an ingredient added in food products, assessment of its stability in the intended food matrices must be performed.^186^ Specific safety considerations with regards to wood hemicelluloses involve, for example, the following items: furans, such as furfural, 5-hydroxymethylfurfural, and furfural diethylacetal, can be formed from pentose or hexose sugars during thermal treatment and oligophenolic compounds from pentose self-condensation.^11,49,187^ The formation of acids, for example, cinnamic acid, abietic acid, and levulinic acid, has also been reported.^11^ While it is possible to reduce the amount of these small molecule contaminants down to trace amounts, it is nevertheless important to consider their safety limits. Additionally, the presence of other minor contaminants and microbial contamination over time are also relevant issues to assess.

The anticipated daily intake of the wood hemicelluloses would vary depending on the intended technical and nutritional function in specific foods. As emulsion stabilizers, the daily intake was estimated to be 32 mg/kg bodyweight/day, which amounts to 2.2 g per person per day, assuming a 70 kg bodyweight. This value is based on the average food consumption of the Finnish population, assuming GGM as the sole emulsifier in dressings, beverages, and margarines;^49^ however, the value may be different from country to country, with regards to the consumption rate of emulsion-based food products. It is less than the intake value of modified and unmodified cellulose (which can have wood origin), which EFSA considers safe up to an intake of 660–900 mg/kg bodyweight/day.^25^ Alternatively, wood hemicelluloses can be further explored as a source of dietary fibers, where a higher level of intake applies. Under EFSA NDA 2010,^188^ dietary fiber is defined as “polysaccharides and lignin that are not digested in the human small intestine”. Dietary fibers are resistant to hydrolysis and absorption in the small intestine and enter the colon substantially unmodified, where they are subjected to fermentation by the colonic microbiota, and, as such, if deemed to demonstrate a positive effect, may be used as a prebiotic; encouragingly, wood hemicelluloses have recently demonstrated such properties, as elaborated on in section 4.1. The present intake of dietary fibers is below the recommended levels at a general population scale (on average 18–24 g/day for adult males and 16–20 g/day for adult females in Europe),^189^ which is lower than EFSA’s recommendation of 25 g/day of dietary fibers to be adequate for normal laxation in adults.^188^

In the U.S., the FDA does not, as yet, recognize novel foods in a separate category. On the basis of the technological aspects described in the previous sections, wood hemicelluloses would be classified as food additives and regulated through the food additive approval process. However, any new food or ingredient can obtain a “generally recognized as safe” (GRAS) designation if a panel of recognized experts affirm that pivotal studies related to the risk assessment of the food under the conditions of its intended use are published in peer-reviewed scientific journals or if the food/ingredient in question was commonly used before 1958. As such, an ingredient is referred to as “self-affirmed GRAS” and voluntarily notified to the FDA.^190,191^ An alternative approach is to petition the FDA to classify the ingredient as a food additive.^192^ In both cases, FDA requires a dossier of risk assessment data similar to that in EU. The key difference between the GRAS and food additive petition is that, in the latter case, data and information on the use of the substance are held privately and sent to the FDA, who establishes the safety of the substance under the intended conditions, in contrast to the GRAS process, where the safety determination is made by qualified experts outside the federal government. Therefore, most companies opt to list new food ingredients as GRAS, when possible, for a rapid premarket approval process.

## 5 Outlook

The growing focus on a circular economy and an increasing need for new food solutions have increased interest in the more efficient usage of wood biomass and application of wood processing side-streams in food instead of disposing them. While forests can be harvested less frequently than crops, they can be harvested all year around and are thus not dependent on seasonal harvesting. Forest biomass is currently used for the production of cellulose and lignin, which leads to low yield and wastage of the prominent hemicellulose fraction. Implementing a wider usage of wood hemicelluloses would provide incentives for the development of new harvesting strategies that could both optimize the use of biomass and preserve the role of forests in the ecosystem. This is highlighted by the fact that there is a vast amount of available wood-based feedstocks that are inadequate for timber/construction material that is still rich in hemicelluloses. Additionally, this volume may rise further due to the spread of wood degrading insects due to the changing climate.^193^ Thus, exploiting wood hemicellulose as food could improve the utilization of wood biomass and contribute to feeding a population that has just passed 8 billion.

We have exhibited in this Review that the current state-of-the-art technological aspects that wood hemicelluloses possess are useful for food applications. Using various extraction methods and postextraction treatments, it is possible to produce hemicelluloses from wood with tailored functionalities for a vast array of food items, ranging from emulsion stabilizers, sources of dietary fiber and probiotics, and even food packaging material. There have also been successful efforts to scale up the extraction process,^194,195^ proving the industrial feasibility of producing wood hemicelluloses. Nevertheless, we acknowledge that there are still several gaps in the knowledge that need to be addressed, such as their behavior in the human digestion process, toxicology, and consumer acceptance. However, vigorous research activities are being conducted to fill those gaps as well as discovering possible new functionalities. Hence, we believe it will not be long before wood hemicelluloses find a way onto kitchen tables around the world.

## Abbreviations Used

Ara: arabinose
AX: arabinoxylan
AGX: arabinoglucuronoxylan
AF4: asymmetric-flow field flow fractionation
DMSO: dimethyl sulfoxide
EC: European Commission
EFSA: European Food Safety Authority
FDA: Food and Drug Administration
Gal: galactose
GalA: galacturonic acid
GalM: galactomannan
GGM: galactoglucomannan
GRAS: generally recognized as safe
GM: glucomannan
GlcA: glucuronic acid
GX: glucuronoxylan
LCC: lignin–carbohydrate complex
MC: methyl cellulose
MDF: microbiota-directed fiber
NDA: panel on nutrition, novel foods, and food allergens
PHWE: pressurized hot-water extraction
TMP: thermomechanical pulp
XG: xyloglucan
